# Interaction of Human Osteoblast-Like Saos-2 and MG-63 Cells with Thermally Oxidized Surfaces of a Titanium-Niobium Alloy

**DOI:** 10.1371/journal.pone.0100475

**Published:** 2014-06-30

**Authors:** Marta Vandrovcova, Ivan Jirka, Katarina Novotna, Vera Lisa, Otakar Frank, Zdenka Kolska, Vladimir Stary, Lucie Bacakova

**Affiliations:** 1 Institute of Physiology, Academy of Sciences of the Czech Republic, Prague, Czech Republic; 2 J. Heyrovsky Institute of Physical Chemistry, Academy of Sciences of the Czech Republic, Prague, Czech Republic; 3 Faculty of Science, J.E. Purkinje University, Usti nad Labem, Czech Republic; 4 Faculty of Mechanical Engineering, Czech Technical University in Prague, Prague, Czech Republic; University of Massachusetts Medical, United States of America

## Abstract

An investigation was made of the adhesion, growth and differentiation of osteoblast-like MG-63 and Saos-2 cells on titanium (Ti) and niobium (Nb) supports and on TiNb alloy with surfaces oxidized at 165°C under hydrothermal conditions and at 600°C in a stream of air. The oxidation mode and the chemical composition of the samples tuned the morphology, topography and distribution of the charge on their surfaces, which enabled us to evaluate the importance of these material characteristics in the interaction of the cells with the sample surface. Numbers of adhered MG-63 and Saos-2 cells correlated with the number of positively-charged (related with the Nb_2_O_5_ phase) and negatively-charged sites (related with the TiO_2_ phase) on the alloy surface. Proliferation of these cells is correlated with the presence of positively-charged (i.e. basic) sites of the Nb_2_O_5_ alloy phase, while cell differentiation is correlated with negatively-charged (acidic) sites of the TiO_2_ alloy phase. The number of charged sites and adhered cells was substantially higher on the alloy sample oxidized at 600°C than on the hydrothermally treated sample at 165°C. The expression values of osteoblast differentiation markers (collagen type I and osteocalcin) were higher for cells grown on the Ti samples than for those grown on the TiNb samples. This was more particularly apparent in the samples treated at 165°C. No considerable immune activation of murine macrophage-like RAW 264.7 cells on the tested samples was found. The secretion of TNF-α by these cells into the cell culture media was much lower than for either cells grown in the presence of bacterial lipopolysaccharide, or untreated control samples. Thus, oxidized Ti and TiNb are both promising materials for bone implantation; TiNb for applications where bone cell proliferation is desirable, and Ti for induction of osteogenic cell differentiation.

## Introduction

Titanium (Ti) - niobium (Nb) alloys have attracted much attention in recent times as promising materials for fabrication of bone implants not only because their non-toxicity, high corrosion resistance and beneficial mechanical properties [1]–[3], but also because of their high biocompatibility, i.e. improved cell adhesion and proliferation, particularly on their oxidized surfaces [4]. The interaction of the cells with the surface of a solid sample is mediated by extracellular matrix (ECM) molecules, which are mainly proteins spontaneously adsorbed to the material surface from biological fluids, such as blood, interstitial fluid or cell culture medium. The cell-material interaction is extensively affected by the conformation of the ECM molecules and their interaction with certain surface sites. As proteins can simultaneously carry charged sites at physiological pH, the surface characteristics related with the surface charge, chemical composition, topography and morphology of the solid sample, are essential for understanding the ECM molecules-surface interaction [4]–[6]. It is a challenging task to discuss the impact of these surface characteristics on the biocompatibility of a solid sample in the course of the development of new biocompatible materials.

The aim of the present work is to describe the interaction between osteoblast-like cells and highly-defined thermally-treated TiNb alloy surfaces, and specifically identify the impact of fine-scale heterogeneity among alloy surface charges on cells that have been seeded onto such surfaces. Two modes of heat-treatment were adopted: in deionized water (dei-H_2_O) under hydrothermal conditions at 165°C, and in a stream of air at 600°C. The first of these two series of samples is referred to below as the Low Temperature (LT) series, while the second is referred to as the High Temperature (HT) series. These dramatically different treatment temperatures should produce oxidized alloy surfaces with substantially different surface characteristics. While the nano-crystalline form of rutile and T-Nb_2_O_5_ oxides together with a portion of amorphous TiO_2_ (Nb_2_O_5_) oxides were found on the surfaces of the samples of HT series [4], other crystalline form(s) of Nb and Ti oxides and a different quotient of their amorphous form might be present in the surfaces of LT samples. The number of charged sites on the surfaces of the samples, i.e. the number of Ti(Nb)-OH groups and defects, and the surface roughness [7], [8] might also be tunable by the oxidation mode. These differences might substantially affect the distribution of the surface charge, acidity and wettability of the samples, i.e. properties frequently used to discuss the interaction of the cells with the surface of materials (cf below). For example, on Ti oxidized at a temperature from 300 to 750°C, the material surface energy, wettability and roughness, measured by the mean surface roughness (R_a_), which reflected the average deviation of the roughness profile of the surface from the mean line, increased with the temperature. Mouse osteoblast progenitor MC3T3-E1 cells cultured on the material reacted to these changes by an increase in the activity of alkaline phosphatase, an early marker of osteogenic cell differentiation [7]. Similarly, thermal oxidation of Ti 530–1000°C led to an increasing content of hydroxyl groups on the material surface and increased formation of calcium phosphate, i.e. a component of the inorganic bone matrix [9].

Thus, increased oxidation temperature seems to induce better osteogenic conditions and better performance of bone-forming cells on pure titanium substrates. The effects of Ti alloys can be more controversial. The Ti-6Al-4V alloy oxidized at 500°C provided a good support for the adhesion, cytoskeletal organization and osteogenic differentiation of human osteoblast hFOB 1.19 cells, while the alloy treated at 800°C behaved as cytotoxic. This was probably due to the enrichment of the alloy surface with vanadium oxides [10]. Similarly, in NiTi alloys, lower oxidation temperatures (400°C) promoted the formation of calcium phosphates on the alloy surface, while higher temperatures (600°C) led to a low rate of Ca and P precipitation, which was explained by a lower content of Ti on these surfaces [11]. In another study, thermal oxidation of Ti-6Al-4V alloy at 700°C enhanced the adhesion of primary human osteoblasts compared with treatment at 500°C only, but transiently impaired the cell proliferation and viability [12], which could be attributed to an increased release of Ti and V ions from the alloy [13].

Thus, it is evident that pure titanium and its alloys can react differentially to the lower and higher oxidation temperatures in terms of their subsequent biocompatibility and the cell behavior. To the best of our knowledge, no study comparing the effects of various thermal oxidation modes on cell behavior on TiNb alloys is available. Our previous study found a beneficial effect of thermal oxidation on the growth of human osteoblast-like MG 63 cells, but it was performed only at a single temperature, i.e. 600°C [4]. In this study, not only two heat-treatment modes, but also two cell models were used, namely MG-63 cells and Saos-2 cells, another human osteoblast-like cell line. Different behavior of these cell lines can be expected, because MG-63 cells are of a less mature and highly proliferative phenotype, while relatively low proliferation activity and high maturation level of Saos-2 cells are closer to the parameters of primary osteoblasts [14]–[16].

This study is focused on (1) a comparison of the adhesion, growth and differentiation of cells in cultures on TiNb alloy, and on cp1-Ti and Nb samples used as standards, (2) a comparison of the cell behavior on these materials after oxidation using two modes of heat-treatment, i.e. hydrothermal conditions at 165°C or a stream of air at 600°C, (3) a comparison of the behavior of bone cells of two different lines, i.e. MG-63 and Saos-2, on the mentioned heat-treated materials, (4) an assessment of the potential cell immune activation by the investigated materials using mouse macrophage-like RAW 264.7 cells, and (5) a correlation of the cell behavior with physicochemical properties of the material surface, namely the concentration of oxygen, charge, topography and morphology.

## Materials and Methods

### Preparation of the samples

TiNb alloy samples, in the form of coupons with mirror-like sheen polished surfaces, were cut from rods used in our previous study [4]. Commercially pure Ti and Nb foils, purchased from Goodfellow Metals, Ltd. (Ti foil: 99.6%, Nb foil: 99.9%) had surfaces polished in the same way as for the alloy samples. All samples were cleaned by sonication in acetone, rinsed in deionized water (dei-H_2_O) and dried in hot air.

The samples of Ti and TiNb of the HT series were oxidized using a previously established method (stream of air, 600°C, 1 hour [4]). The Ti, Nb and TiNb coupons of the LT series were immersed in 40 ml of dei-H_2_O in a Teflon-lined autoclave and heated to 165°C for 12 hours under autogenous pressure. The samples were assigned according to their composition and treatment as: *TiNbx* (*Tix, Nbx*), where *x* stands for temperature of heating (either 165°C or 600°C). The Nb foil was oxidized only by LT treatment, which enabled us to prepare the sample with a compact oxidic layer. Niobium treated at 600°C was covered with unconsolidated grains, which made it impossible to proceed with cellular experiments on the sample, or to characterize it by measuring the ***ζ-***potential and AFM. For these reasons, the Nb foil was only oxidized with the LT treatment, which enabled us to prepare the sample with a compact oxidic layer and proceed with experimental sample characterizations. For further details on preparation of the samples, see Supporting Information S1.

### Characterization of the samples

The chemical composition of the samples was characterized by X-ray photoelectron spectroscopy (XPS), and their crystal structure, morphology and topography were characterized by Raman spectroscopy (RS) and Atomic Force Microscopy (AFM). The surface charge of the samples was measured as the zeta (***ζ-***) potential in an electrokinetic experiment.

The chemical composition of the surfaces of the samples was estimated using an ESCA 3 Mk 2 spectrometer (VG) with a hemispherical analyzer in fixed transmission mode (passing energy of 20 eV). The photoelectrons were excited by the Al Kα_1,2_ emission line (1486.6 eV). The vacuum during each experiment was of the order of ≈10^−9^ mbar. Binding energies E_b_ were calibrated using the E_b_ of the Ti 2p_3/2_ photoelectron line (458.7 eV) [17]. The concentrations *a(x)* of oxygen (***x*** = O), titanium (***x*** = Ti), niobium (***x*** = Nb) and carbon (***x*** = C) of the samples are discussed as atomic ratios: 
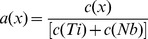
(1)for alloy samples, and 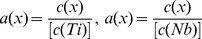
(2)for Ti and Nb samples.

The estimated ***a(O)*** values of the samples were expressed relative to the stoichiometric concentration of oxygen (***a(O)^stechio^***) as ***ΔO***:
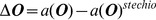
(3)


For further details on the quantitative analysis of the samples, see Supporting Information S2.

The surface morphology was analysed by atomic force microscopy (AFM Dimension ICON, Bruker) operating in air. AFM images were obtained by the PeakForce tapping mode using silicon probes (TESPA, Bruker, USA) with nominal force constant k = 42 N/m and nominal resonant frequency f = 320 kHz. The surface roughness, estimated using AFM line scans (1 µm and 10 µm cut off), was measured as the mean surface roughness (R_a_).

A Labram HR spectrometer (Horiba Jobin-Yvon) interfaced to an Olympus BX-41 microscope was used to measure the Raman spectra used for characterizing the crystal structure of the samples. The spectra were excited by a 633 nm laser, and the laser power under a 50× objective was 2 mW. The Raman spectrometer was calibrated using the F1g line of Si at 520.5 cm^−1^.

A SurPASS Instrument (Anton Paar, Graz, Austria) was used to measure the electrokinetic ζ- potentials. The samples were studied inside the adjustable gap cell in contact with the electrolyte (10^−3^ mol·dm^−3^ KCl) at the physiological pH value (7.4). The relative experimental error was 10%. Further details of the electrokinetic experiment can be found in [18].

### Cell seeding

For cell culture experiments, samples were sterilized under UV light, inserted into 12-well polystyrene cell culture plates (TPP, Switzerland; internal well diameter 21.4 mm) and seeded with: i) human osteoblast-like MG-63 cells (European Collection of Cell Cultures, Salisbury, UK; Cat. No. 86051601) suspended in Dulbecco's modified Eagle's Minimum Essential Medium (DMEM; Sigma, USA, Cat. No. D5648), ii) Saos-2 cells (European Collection of Cell Cultures, Salisbury, UK, Cat. No. 89050205) suspended in McCoy medium (Sigma, Cat. No. M4892), or iii) murine macrophage-like RAW 264.7 cells (ATCC, TIB-71, USA) suspended in RPMI-1640 medium (Sigma, Cat. No. R8758). All culture media were supplemented with 10% fetal bovine serum (Sebak GmbH, Aidenbach), and gentamicin (40 µg/mL, LEK). Each well contained i) 36,000 MG-63 cells (i.e., approximately 10,000 cells/cm^2^), ii) 20,000 Saos-2 cells (5,600 cells/cm^2^) or iii) 50,000 RAW 264.7 cells (14,000 cells/cm^2^), and 2 mL of the medium. The cells were cultured for 1 and 3 days (to evaluate cell numbers) or for 7 days (for PCR detection of markers of osteoblast differentiation, and to measure the potential immune activation of RAW cells) at 37°C in a humidified air atmosphere containing 5% CO_2_. Two samples were used for each experimental group and time interval.

### Evaluating the cell numbers

On day 1 after seeding, the samples were rinsed with phosphate-buffered saline (PBS; Sigma, Cat. No. P4417), fixed with 70% ethanol at room temperature for 20 minutes, and stained with a combination of two fluorescence dyes: Texas Red C_2_-maleimide cell membrane dye (Molecular Probes, Invitrogen, Cat. No. T6008; 20 ng/ml), and Hoechst #33258 cell nucleus dye (Sigma, Cat. No. B1155; 5 µg/ml) for 1 hour at room temperature. The number of cells on the material surface was evaluated on microphotographs taken under an Olympus IX-51 microscope, equipped with an Olympus DP 70 digital camera.

On day 3 after seeding, the number of cells was evaluated after immunofluorescence staining of **vinculin** (protein of focal adhesion plaques, participating in cell–substrate adhesion and stabilizing the focal adhesions), and **β-actin** (an important component of the cell cytoplasmic cytoskeleton, associated with focal adhesion plaques). The staining has been described in detail in [5], and is also described in Supporting Information S3.

### Quantitative real-time PCR (q-PCR)

Q-PCR was used to determine the effect of the material surface chemistry on the levels of gene expression for: collagen type I (COL1; the predominant component of the bone ECM during osteoblast maturation, participating in the mineralization process), alkaline phosphatase (ALP; an enzyme involved in bone tissue mineralization), and osteocalcin (bone gamma-carboxyglutamic acid-containing protein, BGLAP; non-collagenous calcium-binding protein of the bone ECM). Further characteristics of these markers are provided in Supporting Information S4.

Saos-2 and MG-63 cells were grown on the tested materials for 7 days. Total RNA was extracted from cultures using RNAzol reagent (Molecular Research Center, inc., Cat. No. RN190) according to the manufacturer's protocol. The mRNA concentration was measured using a NanoPhotometer S/N (IMPLEN), and extracted RNA was stored at −70°C before reverse transcription. Reverse transcription was performed on 1 µg of total RNA using the ProtoScript M-MuLV First Strand cDNA Synthesis kit (New England BioLabs, Cat. No. E6300S) according to the manufacturer's protocol, and a T Personal Therocycler (Biometra). Q-PCR primers were purchased from Generi Biotech s.r.o. (Table 1).

**Table 1:** Oligonucleotide primers for q-PCR amplifications.

Gene	Primers sequence	Product size (bp)
***Osteocalcin***		70
	Forward: 5′-GAAGCCCAGCGGTGCA-3′	
	Reverse: 5′-CACTACCTCGCTGCCCTCC-3′	
***ALP***		68
	Forward: 5′-GACCCTTGACCCCCACAAT-3′	
	Reverse: 5′-GCTCGTACTGCATGTCCCCT-3′	
***Collagen I***		83
	Forward: 5′-CAGCCGCTTCACCTACAGC-3′	
	Reverse: 5′-TTTTGTATTCAATCACTGTCTTGCC-3′	
***GAPDH***		87
	Forward: 5′-TGCACCACCAACTGCTTAGC-3′	
	Reverse: 5′-GGCATGGACTGTGGTCATGAG-3′	

In addition, a primer specific for the endogenous gene GAPDH was used as a so-called housekeeping gene in order to normalize expression levels. Real-time PCR was performed using an iCycler (iQ 5 Multicolor Real-Time PCR Detection System, Bio-Rad). Each real-time PCR reaction was performed with the following reagents: 10 µl SYBR green (Roche, Cat. No. 04913914001), 0.2 µl each of sense and antisense primer, 2 µl of reverse transcription product, and an appropriate volume of nuclease-free water to bring the total reaction volume to 20 µl. Duplicate PCR reactions were prepared for each sample at the following thermal cycling conditions: initiation at 95°C for 10 min, then 40 cycles were performed, each of them consisting of denaturation at 95°C for 15 s and hybridization-elongation at 60°C for 1 min. The point at which the PCR product was first detected above a fixed threshold (termed cycle threshold, *C*_t_), was determined for each sample. Changes in the expression of the target genes were calculated using 2^−ΔΔ*Ct*^, where ΔΔ*C_t_* was calculated from Equation (3), taking samples of MG-63 or Saos-2 cells grown on a tissue culture polystyrene dish as a calibrator.
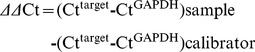
(4)


### Immune activation of RAW 264.7 cells

The potential immune activation of cells was estimated by the concentration of tumor necrosis factor alpha (TNF-α) in the cell culture media after cultivation of RAW 264.7 cells on the tested materials and polystyrene (PS) control dishes for 7 days. Measurements were performed using the commercially available Mouse TNF-α ELISA Kit (Thermo Scientific Inc., Rockford, IL, USA, Cat. No. 1347.4 EMTNFA) in accordance with the manufacturer's protocol. This procedure has been described in detail in [19], and is also provided in the Supporting Information S5.

### Statistical analysis

The cell number on microphotographs on day 1 and 3 after seeding was presented as mean ± S.E.M. (Standard Error of Mean) from 32 measurements. The values of the ζ-potentials were expressed as mean ± S.E.M. from 2 measurements. The amount of TNF-α was presented as mean ± S.E.M. from 4 measurements. PCR data were presented as mean ± S.D. (Standard Deviation) from 2 measurements. Multiple comparison procedures were performed with ANOVA, using the Student–Newman–Keuls method. A value of *P*≤0.05 was considered significant.

## Results

### Physicochemical properties of the samples

XPS analysis of the samples revealed that Ti is present on the surfaces of the samples solely as TiO_2_, and Nb as Nb_2_O_5_ (Table 2). The value of ***a(Nb)*** of samples *TiNb165* (0.37) and *TiNb600* (0.33) were at least qualitatively similar. The estimated values of ***Δ(O)*** vary significantly (from −0.31 for *Nb165* to 0.70 for *Ti165*, cf. Table 2) along the series of investigated samples. They reflect the concentration of skeletal O atoms of the oxides and surface −OH groups created by dissociation of Ti-O-Ti (Nb-O-Nb) bonds modulated by the presence of defective sites with a missing O atom in the lattice. The presence of −OH groups in the samples caused an increase in the ***a(O)*** value of the oxides, while the presence of defective sites in the samples caused a decrease in ***a(O)***. The overall distribution of these sites was strongly affected by the mode of heat treatment and by the chemical composition of the sample surface (cf. Table 2).

**Table 2:** Summary of results obtained in characterization of Ti, Nb and TiNb samples treated at 165°C or 600°C.

	165°C	600°C	Method
***Morphology***			AFM, XPS
TiNb	grains 50–160 nm	amorphous phase on top	
Ti	grains 10–70 nm	amorphous phase on top	
Nb	structure-less		
***Cryst. Struct.***			Raman
TiNb	anatase + Nb_2_O_5_ (amorph.)	rutile +T-Nb_2_O_5_	
Ti	anatase	rutile	
Nb	amorphous	T-Nb_2_O_5_	
***R_a_*** [nm])			AFM
TiNb	5.7 (5.75)^a^	5.2 (1.4)	
Ti	14.3 (6.5)	13.3 (9.9)	
Nb	3.1 (0.9)	-	
***ζ*** [mV]			Electrokin. exp.
TiNb	−45.4±4.37	−48.0±1.5	
Ti	−51.1±2.96	−60.0±3.00	
Nb	−48.3±3.19	-	
***c(Nb)***			XPS
TiNb	0.37	0.33	
***ΔO***			
TiNb	0.05	0.25	
Ti	0.70	0.50	
Nb	−0.31		
***c(C)***			
TiNb	0.93 (0.21)^b^	0.72 (0.30)	
Ti	0.68 (0.11)	1.05 (0.41)	
Nb	0.65 (0.20)	-	

a estimated on 10 µm cut off, numbers in brackets: 1 µm cut off.

b overall concentration of C, numbers in brackets concentration of oxidized C species.

***R_a_***: mean surface roughness.

***ζ***: zeta potential.

***c(Nb), c(C), ΔO***: concentration of niobium, carbon and relative concentration of oxygen, respectively.

The values of the ζ-potential were expressed as mean ± S.E.M. from 2 measurements.

**Figure 1:**
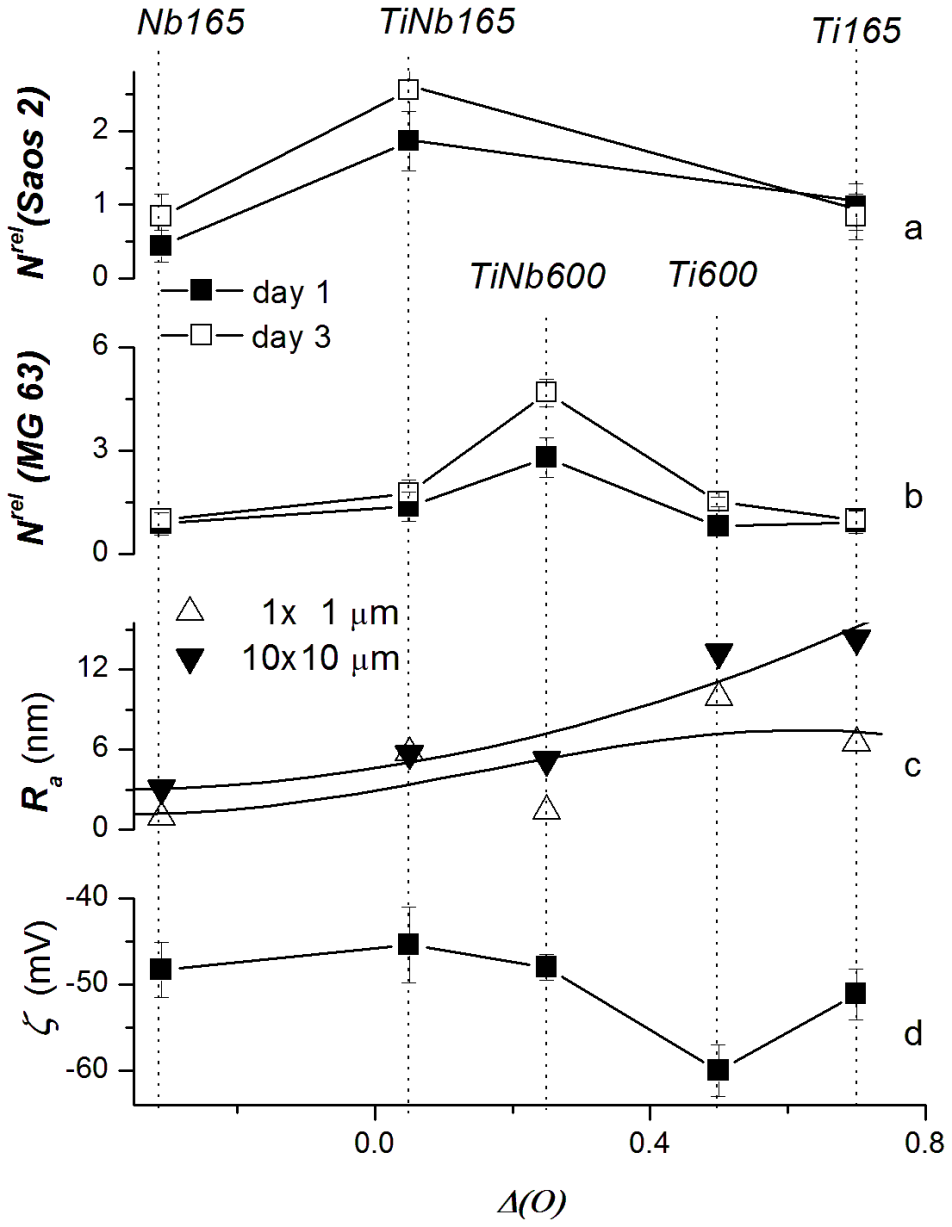
Correlation of relative numbers of human osteoblast-like Saos-2 and MG-63 cells, i.e. *N^rel^(Saos 2)* (a), *N^rel^(MG 63)* (b), mean surface roughness *R_a_* (1 and 10 µm cut off) (c) and zeta (*ς*-) potential (d) with the relative concentration of oxygen *Δc(O)* on the surface of Ti, Nb and TiNb samples treated at 165°C or 600°C. Experiments on day 1 and day 3 are graphically distinguished. Data are presented as mean ± S.E.M. from 32 measurements (cell number) or 2 measurements (ζ- potential) for each experimental group and time interval.

**Figure 2:**
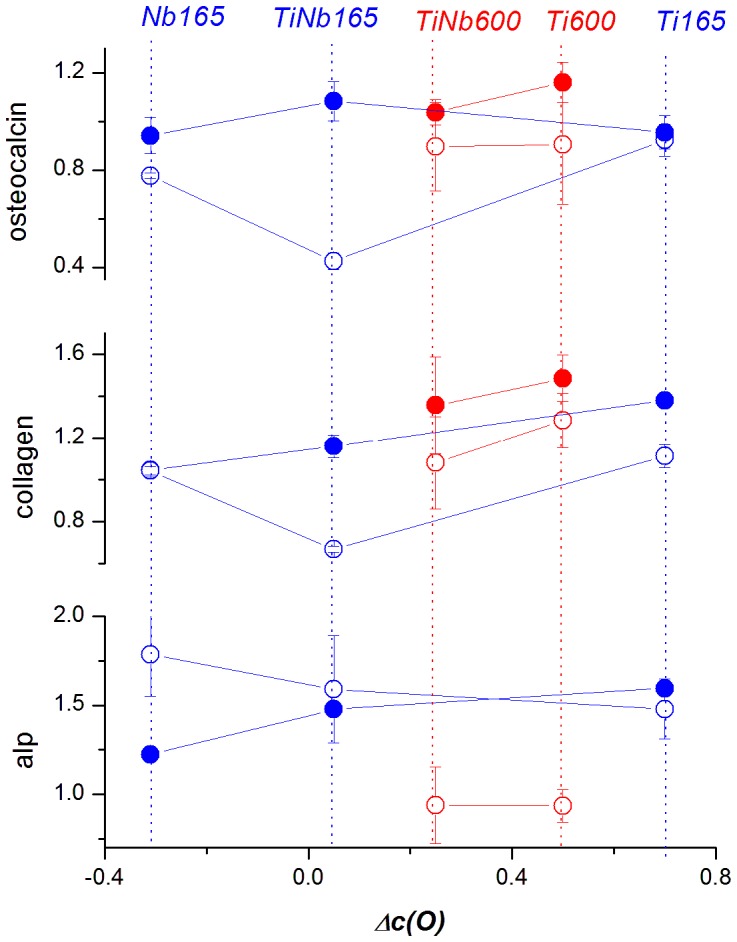
Correlation of the expression of specific genes of MG-63 cells (full symbols) and Saos-2 cells (open symbols) with the relative concentration of oxygen *Δc(O)* on the surface of Ti, Nb and TiNb samples treated at 165°C (blue) or 600°C. Mean ± S.D. from 2 measurements for each experimental group.

The presence of the –OH groups and defective sites can affect the line shape of photoelectron O 1 s spectra. However, a rather high concentration of oxidized hydrocarbons disabled a line shape analysis of the O 1 s photoelectron spectra for investigated samples (cf. concentration of oxidized C in Table 2). Evaluated values of ***Δ(O)***, corrected for this contamination (cf. Supporting Information S2), were used as a suitable correlation parameter to simultaneously compare the effect of surface roughness ***R_a_*** and ***ζ-***potential on the colonization of material surfaces by cells along the series of samples (Figure 1), and also on the expression level of specific differentiation genes by the cells (Figure 2).

**Figure 3:**
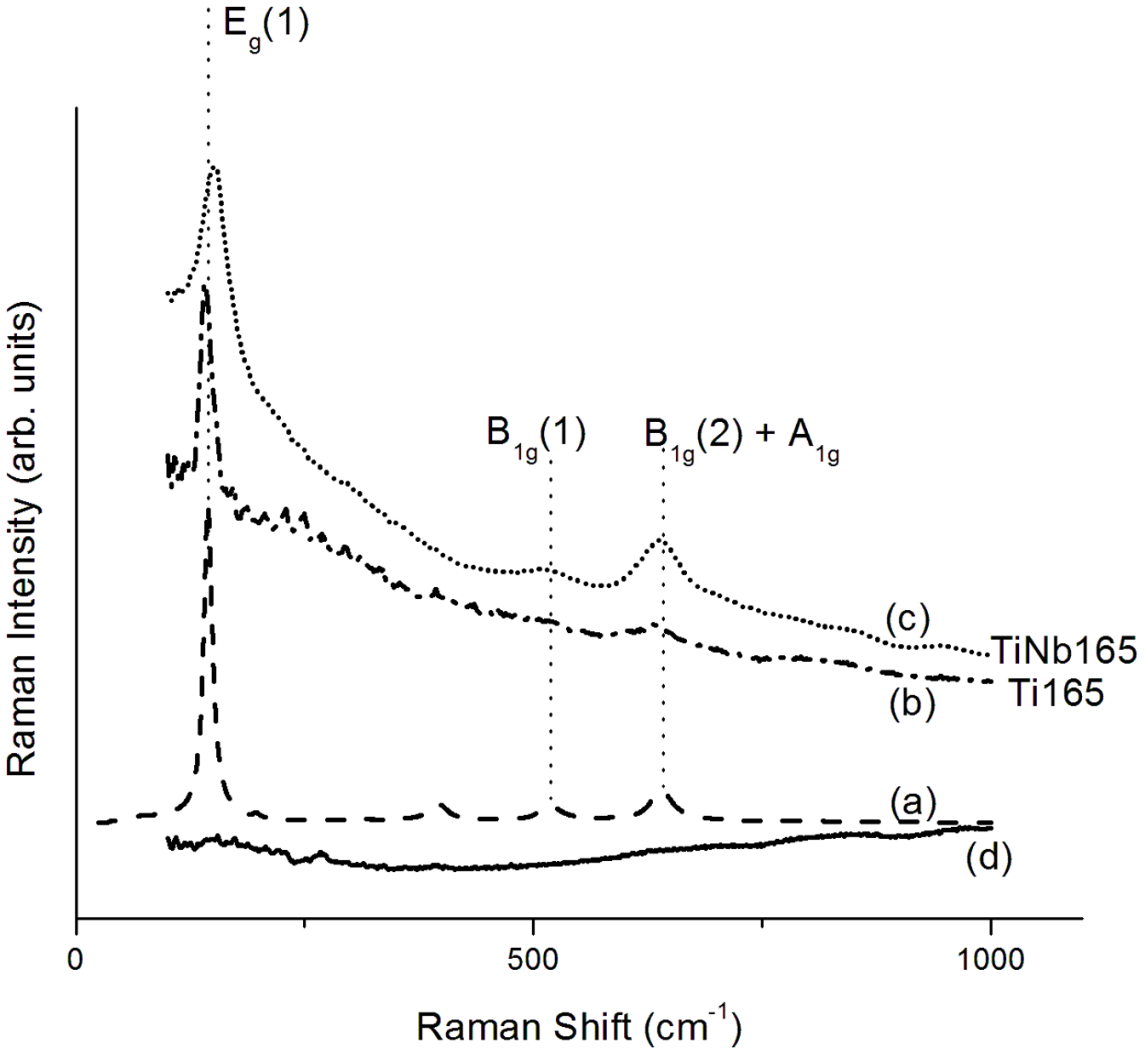
Raman spectra of anatase (a) and metallic samples treated at 165°C, referred to as *Ti165* (b) *TiNb165* (c) and *Nb165* (d).

Figure 3 compares the Raman spectra of the LT samples with the spectrum of anatase. Three bands (E_g_(1), B_1g_(2)+A_1g_ and E_g_(1) vibrations) and two bands (E_g_(1) and E_g_(3) vibrations) present in the Raman spectrum of anatase were found in the spectrum of the *TiNb165* and *Ti165* samples, respectively. However, no Raman spectrum was found for sample *Nb165*. Thus, the titanium oxide phase was present in samples *Ti165* and *TiNb165* in the form of anatase. As no Raman spectrum is observed for Nb oxide phase in the LT samples (Figure 3) this phase is related to the amorphous one in samples *Nb165* and *TiNb165*. The Ti and Nb oxides were identified by XPS as Nb_2_O_5_ and TiO_2_. The Ti phase of *TiNb600* and *Ti 600* is rutile, and the Nb phase of the HT alloy sample is T-Nb_2_O_5_ with some portion of amorphous Ti (Nb) oxide phases [4]. No evidence for the presence of Ti-Nb mixed oxide phase is obtained from the Raman and XPS spectra of the HT alloy samples [4] and the LT alloy samples.

**Figure 4:**
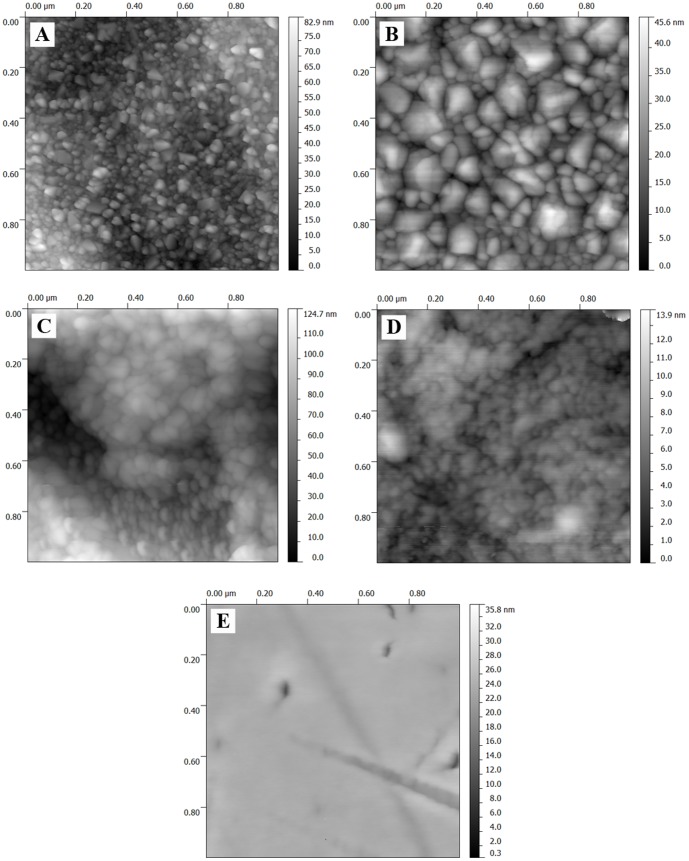
Atomic force microscopy (AFM) images (1×1 µm) of metallic samples treated at 165°C or 600°C, namely *Ti165* (A), *TiNb165* (B), *Ti600* (C), *TiNb600* (D), and *Nb165* (E).

The AFM images (1×1 µm) summarized in Figure 4 were used for depicting the variability of the sample morphology. Specifically, the surfaces of the *Ti165* and *TiNb165* samples were nano-structuralized, i.e. covered with grains of irregular sizes and dimensions ∼10–∼70 nm (*Ti165*) and ∼50–∼160 nm (*TiNb165*). On the surfaces of the HT samples, a suppressed nano-structure was observed. In particular, the surface of sample *Nb165* is entirely structure-less. Suppressed nano-structure of the HT samples in comparison with the LT samples was also observed by Scanning Electron microscopy (not shown here).

Surface topography, measured as the mean surface roughness ***R_a_*** estimated from the 1×1 µm and 10×10 µm AFM images, is defined as the average deviation of the roughness profile from the mean line (for a review dealing with the effect of ***R_a_*** on biocompatibility, see [5], [20]). The values of ***R_a_*** are summarized in Table 2. Estimated values of ***R_a_*** are in the range of 3.1–14.3 nm (cut off 10 µm) and 0.9–9.9 nm (cut off 1 µm) along the series of investigated samples. The dependence of the ***R_a_*** values on ***Δc(O)*** is depicted in Figure 1c.

The ***ζ-***potential measurements of LT and HT samples (summarized in Table 2) are depicted against ***Δ(O)*** in Figure 1d. The values of the ***ζ-***potentials of samples *Ti165* (−51.1±2.96 mV) and *Ti600* (−60.0±3.00 mV) were systematically lower than the values of the ***ζ-***potentials of samples *TiNb165* (−45.4±4.37 mV) and *TiNb600* (−48.0±1.5 mV). The ***ζ-***potentials of the alloy samples and sample *Nb165* (***ζ*** = 48.3±3.19 mV) are the same within the experimental error. The ***ζ-***potentials for the HT series of samples were adopted from our earlier study [4].

### Cell numbers

To facilitate a comparison of all data used in the following discussion, the ***N(x)*** values are normalized relative to the number of cells estimated for a polystyrene dish (***N(PS)***) in the same experiment, and assigned as ***N(x)^rel^*** *-* ***Z*** (***Z*** =  abbreviation of the relevant sample) (Tables 3, 4).

**Table 3:** The relative values of cell numbers, i.e. ***N^rel^(x)***(***x*** =  ***Saos-2***, ***MG-63***), measured on days 1 and 3 after seeding of Saos-2 or MG-63 cells on Ti, Nb and TiNb samples treated at 165°C or 600°C.

Sample	*N^rel^(Saos-2)*	*N^rel^(MG-63)*
*Ti165*	0.97±0.32 (0.84±0.31)^a^	0.93±0.32 (1.00±0.29)
*TiNb165*	1.86±0.40***^Nb^*** (2.55±0.55***^Ti, Nb, PS^***)	1.38±0.42 (1.77±0.38)
*Nb165*	0.44±0.22 (0.84±0.3)	0.88±0.32 (1.00±0.30)
*Ti600*	not analysed	0.80±0.23 (1.51±0.14)
*TiNb600*	not analysed	2.80±0.58***^Ti, 165, PS^*** (4.69±0.40***^Ti, 165, PS^***)
*PS*	1.00±0.07 (1.00±0.10)	1.00±0.10 (1.00±0.09)

a numbers in bracket: day 3 after seeding the cells. Mean ± S.E.M. from 32 measurements for each experimental group and time interval. ANOVA, Student-Newman-Keuls Method. Statistical significance: p≤0.05 compared to the value on reference Ti or Nb samples treated at a corresponding temperature (**^Ti, Nb^**), on corresponding LT samples (**^165^**), and on polystyrene dishes (**^PS^**).

**Table 4:** Absolute values of the cell population density (cells/cm^2^) of Saos-2 and MG-63 cells measured on a polystyrene dish ***N(PS)*** in the series of experiments with samples heat-treated at 165°C and 600°C used for creation of ***N^rel^(x)*** in Table 3.

Sample series	Saos-2, day 1	Saos-2, day 3	MG-63, day 1	MG-63, day 3
165°C	4800±300	7670±855	21800±1790	123209±8570
600°C	not analysed	not analysed	10270±1020	33000±2920

Mean ± S.E.M. from 32 measurements for each experimental group and time interval.

The initial adhesion of Saos-2 and MG-63 cells on day 1 after seeding on the samples increased as follows:
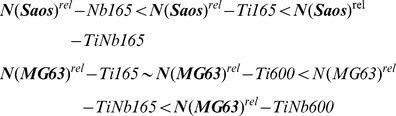



The ***N(MG63)*** values of samples *Ti600* and *TiNb600* were adopted from our previous study [4]. The highest initial adhesion of MG-63 cells and Saos-2 cells on day 1 was observed on the TiNb alloy samples. On samples treated at 165°C, the value of ***N^rel^(Saos-2)*** on TiNb was 1.86±0.40, while these values for samples *Ti165* and *Nb165* were only 0.97±0.32 and 0.44±0.22, respectively. Similarly, the value of ***N^rel^(MG-63)*** on TiNb was 1.38±0.42, while the values for *Ti165* and *Nb165* were only 0.93±0.32 and 0.88±0.32, respectively. On samples treated at 600°C, the ***N^rel^(MG-63)*** value on TiNb was 2.80±0.58, while on Ti it was only 0.80±0.23. When samples treated at different temperatures were compared, the ***N^rel^(MG-63)*** value on samples *TiNb165* (1.38±0.42) was much lower than on samples *TiNb600* (2.80±0.58), while the values on *Ti165* (0.93±0.32) and *Ti600* (0.80±0.23) were similar (Table 3, Figures 1a, b).

The values of ***N(x)^rel^*** *-Z* increased further on day 3 after seeding. The order of the ***N(x)^rel^*** *-Z* values remained the same as on day 1, with the exception of the values of ***N(Saos)^rel^*** *-Nb165* and ***N(Saos)^rel^*** *-Ti165*, which became equal. Similarly as on day 1, the increase in ***N^rel^(MG63)*** on sample *TiNb165* was much less pronounced than on sample *TiNb600*. ***N^rel^(MG63)^rel^*** *-* ***TiNb600*** had a greater effect on cell growth than all other ***N(x)^rel^*** *-* ***Z***. On day 3, the number of MG-63 cells on *TiNb600* was 4.69 times higher than on polystyrene, while on *Ti600* the number was only 1.51 times higher. In samples treated at 165°C, this value amounted only to 1.77 in TiNb and 1.00 in Ti (Table 3, Figures 1a, b).

Although the MG-63 cells generally reached much higher cell population densities than the Saos-2 cells, the differences in cell numbers between samples Ti165°C and TiNb165°C were more apparent in the Saos-2 cells. More specifically, on day 3 after seeding, the number of Saos-2 cells on the TiNb samples, normalized to polystyrene, was 2.55 compared to 0.84 on Ti, while in the MG-63 cells the ratio was only 1.77 *vs*. 1.00 (Table 3).

The cell number increased with increasing ***Δ(O)*** in samples with lower surface roughness (Nb, TiNb). On samples with higher surface roughness (Ti), the cell number declined, although these samples showed the highest ***Δ(O)*** values, i.e. the highest excess of O concentration above the stoichiometric value (Figure 1). In addition, the number of cells was higher on TiNb samples characterized with less negative ζ-potential than on Ti samples with more negative ζ-potential. Specifically, on TiNb samples (ζ-potential from −45.4±4.37 to −48.0±1.5), the relative cell number ranged from 1.38±0.42 to 4.69±0.40, while on Ti samples (ζ-potential from −51.1±2.96 to −60.0±3.00), the cell number was only between 0.80±0.2 and 1.51±0.14 (Figure 1, Tables 2 and 3).

### Cell morphology

**Figure 5:**
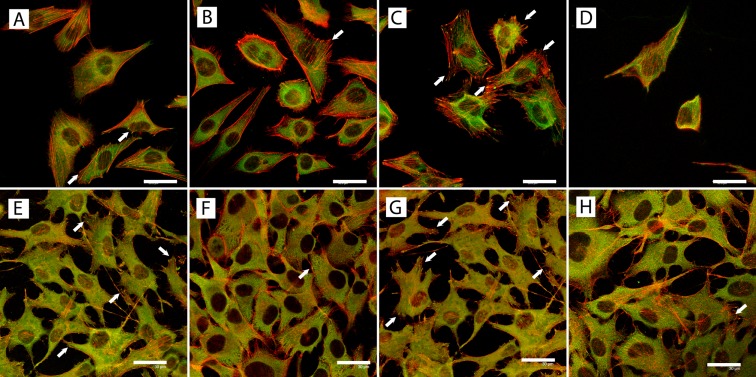
Immunofluorescence staining of vinculin (green) and beta-actin (red) in Saos-2 cells (A–D) and MG-63 cells (E-H) on day 3 after seeding on metallic samples treated at 165°C, i.e. *Ti165* (A, E), *TiNb165* (B, F) and *Nb165* (C, G), and a microscopic glass coverslip (D, H). Arrows indicate vinculin-containing focal adhesion plaques. Leica SP2 confocal microscope, Germany. Bar  = 30 µm.

Cell morphology was evaluated only in cells on samples heated to 165°C (Figure 5). The Saos-2 cells were relatively sparse, and better reflected the cell numbers calculated on the tested samples from the microphotographs than the MG-63 cells. The Saos-2 cells were more numerous on the TiNb samples than on pure Ti or Nb, and also on microscopic glass coverslips. At the same time, the MG-63 cells appeared to be near confluence on all tested samples. Both cell types were mostly well-spread and polygonal with long processes. This was apparent particularly in MG-63 cells (Figure 5).

The cells on all tested samples showed positive staining for β-actin and vinculin. The β-actin cytoskeleton was better developed in the Saos-2 cells, i.e., β-actin was assembled into distinct filaments, while it was distributed rather diffusely in the MG-63 cells. The β-actin-containing filaments were well apparent particularly at the cell periphery, while vinculin was localized rather centrally. Vinculin was distributed mostly diffusely, but at the cell periphery distinct focal adhesion plaques co-localizing with actin filaments were also apparent in both MG-63 and Saos-2 cells, particularly in cultures on pure Ti or pure Nb.

### Cell viability

The viability of the cells on all tested materials, evaluated by the LIVE/DEAD viability/cytotoxicity kit, was very high, nearly 100%, and there were no significant differences among the tested groups (data not shown here).

### Expression of specific genes

**Figure 6:**
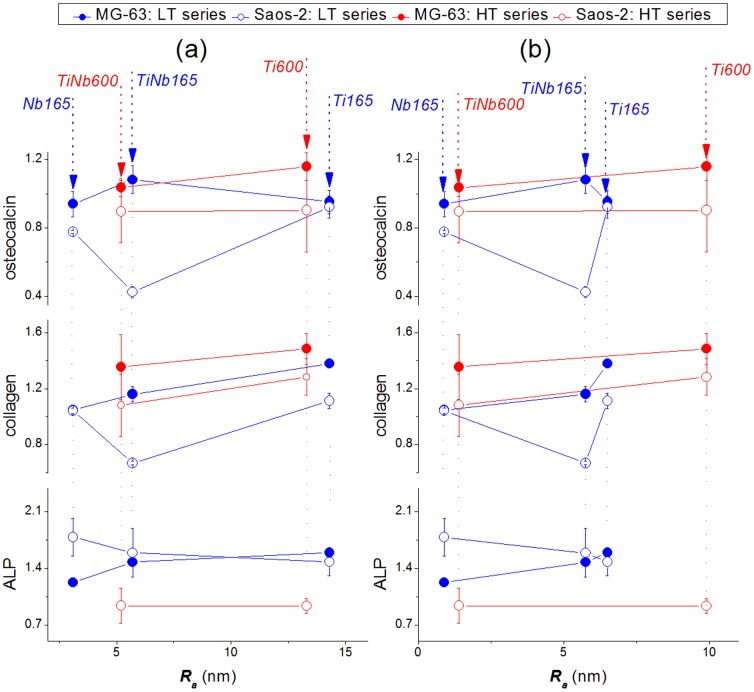
Correlation of the expression of specific genes of MG-63 and Saos-2 cells on Ti, Nb and TiNb samples of the High Temperature (HT, 600°C) and the Low Temperature (LT, 165°C) series with mean surface roughness (*R_a_*) values estimated at 1 µm (a) and 10 µm (b) cut off. Mean ± S.D. from 2 measurements for each experimental group.

The correlations of the expression of specific genes in the MG-63 and Saos-2 cells with ***Δc(O)*** and with ***R_a_*** in the material samples are depicted in Figures 2 and 6. After 7 days of cultivation, the MG-63 cells had very low levels of mRNA for ALP, which were at the limit of detection (Table S1). Nevertheless, the levels of mRNA for collagen I and osteocalcin in MG-63 cells on the tested materials were similar to the values in cells grown on PS dishes, and thus sufficiently high for the PCR analysis. These values did not differ significantly among the tested samples. The relative values of mRNA expression on the tested metallic samples, normalized to the values obtained in cells on PS, ranged from 0.941±0.074 to 1.160±0.083 for osteocalcin, and from 1.042±0.140 to 1.484±0.111 for collagen I (Table S2). In the case of collagen I, gene expression values tended to increase with increasing ***Δc(O)***, ***R_a_*** and also with the treatment temperature (Figures 2, 6).

The differences in osteogenic gene expression in the cells on the tested samples became more apparent when Saos-2 cells were used. The correlation of the gene expression with the value of ***Δc(O)*** is depicted in Figure 2. The expression level of osteocalcin and collagen I was lowered in the cells on the *TiNb165* sample relative to the expression of all other samples, including the *Ti165* samples and the HT samples. On the other hand, the expression of ALP was lowered for the samples in the HT series.

Correlations of the expression of differentiation genes with the value of ***R_a_*** estimated from AFM images (cut off 1 and 10 µm) are depicted in Figure 6a, b. It follows from this Figure that the observed lowered expression of osteocalcin and collagen in Saos-2 cells on sample *TiNb165* corresponds to the value ***R_a_*** = 5.7 nm estimated from the 10 µm cut off and 5.75 nm from the 1 µm cut off. However, the observed higher expression of osteocalcin and collagen on the surfaces of all other samples do not correlate with ***R_a_*** (Figure 6). For example, in Saos-2 cells on the LT samples, the expression of osteocalcin and collagen is similar in cells on samples with the lowest ***R_a_*** (Nb) and with the highest ***R_a_*** (Ti). On the other hand, the expression of osteocalcin and collagen in Saos-2 cells on the LT samples was markedly higher on Ti than on TiNb, i.e., on rougher samples than on smoother samples. On Ti, the level of mRNA for osteocalcin and collagen was 0.923±0.066 and 1.113±0.056, respectively, while on TiNb, it was only 0.425±0.0331 and 0.667±0.0139 (Table S2). The expression of ALP (systematically higher on the surfaces of the LT samples) also does not correlate with ***R_a_*** (Figure 6).

### Secretion of TNF-α

The production of TNF-α by RAW 264.7 cells was measured only on samples treated at 165°C. The concentration of TNF-α in the cell culture medium was similar in all three heat-treated samples and standard cell culture polystyrene dishes. At the same time, these concentrations were significantly lower than in the case of non-treated metallic samples, and also lower by 1 to 2 orders than after stimulation of RAW 264.7 cells with bacterial LPS (Table 5).

**Table 5:** Amount of TNF-α in the culture medium (pg/million cells) produced by cells cultivated on Ti, Nb and TiNb modified at 165°C or 600°C, or treated by various concentrations of bacterial lipopolysaccharide (LPS).

Value	Ti non- treated	TiNb non- treated	Ti 165°C	Nb 165°C	TiNb 165°C	PS (no LPS)	1 ng/mL LPS	10 ng/mL LPS	50 ng/mL LPS
Mean	73.6*	84.0*	53.4	56.5	55.5	52.7	767.1*	1374.1*	2097.1*
S.E.M.	0.5	3.2	1.3	1.2	0.5	1.5	24.2	134.3	75.3

Mean ± S.E.M. from 4 measurements for each experimental group (on day 7 after seeding). ANOVA, Student-Newman-Keuls Method. Asterisks indicate experimental groups significantly differing (p≤0.05) in TNF-α production from heat-treated metallic samples.

## Discussion

### Cell adhesion and growth on tested samples

The first finding of our study was that the number of human osteoblast-like MG-63 and Saos-2 cells on days 1 and 3 after seeding was higher on TiNb than on the Ti and Nb samples. While the MG-63 cell numbers on the Ti and Nb samples on day 1 after seeding were similar to the values on the polystyrene dishes, the numbers on TiNb were 1.4 (LT series) to 2.8 times higher (HT series, [4]) than on polystyrene. A further increase in the number of MG-63 cells proceeded on day 3 after seeding. Similarly, the number of Saos-2 cells on sample *TiNb165* was also higher both on day 1 and on day 3 after seeding than on the polystyrene dish and on the *Ti165* and *Nb165* samples (Figure 1, Table 3). Thus, the presence of Nb_2_O_5_ phase in the alloy sample stimulates the initial adhesion and subsequent growth of cells on the material surface.

A positive effect of an Nb-containing surface phase on the interaction of the material with the cells has also been observed by others. Zhao *et al.* [21] compared the proliferation of human osteoblast cells on commercial cp-Ti discs coated with TiO_2_, TiO_2_-Nb_2_O_5_ and TiO_2_-SiO_2_. The highest proliferation was observed for an Nb_2_O_5_-containing coating. Increased adhesion and spreading of human mesenchymal stromal cells on the Piranha etched surface of a *Ti40Nb* alloy containing an Nb_2_O_5_ phase relative to the adhesion and spreading of the cells on the etched surface of Ti with the same surface topography has recently been observed [22]. In line with our previous results [4], the increased biocompatibility of the alloy surface was attributed to the presence of the surface Nb_2_O_5_ phase, which strongly affects the surface energy and the charge of the alloy. To test the role of the surface charge in the samples in our study, a discussion of other effects, including surface morphology, topography and crystal structure should be included.

### The role of material surface morphology, roughness and crystal structure in cell-material interaction

The effect of the structural differences of samples *TiNb165* and *Nb165* on the adhesion and proliferation of the cells on their surfaces is rather straightforward. The *Nb165* samples did not increase the number of initially adhering cells or their subsequent growth, i.e. the cell numbers on this material remained similar to or even lower than the numbers on polystyrene (Table 4, Figure 1). According to AFM (Figure 4, values of ***R_a_***, Table 2), and Raman spectroscopy (Figure 3), the surface of this material is the smoothest among all investigated samples, and is entirely amorphous and structure-less. It is known that completely structure-less substrates are less appropriate for cell adhesion and growth than nano-structured materials [23], [24]. The nanostructure of the cell growth substrate has repeatedly been shown to have a stimulatory effect on cell adhesion and subsequent growth (e.g. [5], [21]). It is believed that on nano-structured substrates with irregularities of certain characteristic dimensions, cell adhesion-mediating proteins, such as vitronectin and fibronectin, present in the serum supplement of cell culture media, are adsorbed in an appropriate and more physiological geometrical conformation, which makes them well accessible for cell adhesion receptors. In addition, nano-structured surfaces adsorb preferentially vitronectin, because its molecules are smaller and less complex (i.e., linear) than other ECM molecules. Vitronectin is then recognized by the adhesion receptors on osteoblasts through the sequence Lys-Arg-Ser-Arg (KRSR) present in the vitronectin molecule [24], [25]; for a review, see [26]. In accordance with these findings, the nano-structural character of a *TiNb165* surface composed from grains with characteristic dimensions 50–160 nm with partial crystallinity (TiO_2_ phase in a form of anatase) was associated with higher colonization of the surface by cells than in the case of colonization on the amorphous and structure-less surface of sample *Nb165*.

The effect of the structural differences of samples *TiNb165* and *Ti165* on the adhesion and proliferation of cells on their surfaces is less clear. Smaller grain dimensions of the sample were found in sample *Ti165* (10–70 nm) than in *TiNb165* (50–160 nm), see Figure 4. The observed morphology of the *Ti165* sample might better correspond to the irregularities on the cell membrane (for example, the length of the extracellular parts of the integrin adhesion receptors, which is about 2 nm) [27]. Similarly, the adhesion of vascular endothelial cells to polymeric surfaces containing nanohills of 13 to 95 nm was most pronounced on surfaces with the lowest nanohills [28]. Similarly, human osteoblasts cultured on surfaces with pits 14 nm and 29 nm in depth showed higher cell attachment, spreading area, expression of integrins and synthesis of focal adhesion proteins than cells on surfaces with pits 45 nm in depth [23]. Thus, the observed higher adhesion and proliferation of the cells on the surface of sample *TiNb165* than on sample *Ti165* may not be dominantly affected by nano-structural effects.

In spite of the larger grain size, the *TiNb165* sample displayed lower surface roughness, measured by the ***R_a_*** parameter (Table 2, Figure 1c). This means that both TiNb and Ti surfaces of the LT series are wavy and irregular (i.e. they contain prominences and pits) yet the amplitude of these irregularities is lower in the *TiNb165* samples than in the *Ti165* samples. Thus, the deformation of the cytoplasmic membrane of adhered cells and their membrane tension can be lower on *TiNb165* than on *Ti165*, and the *TiNb165* sample may provide better conditions for cell adhesion and growth.

The nanostructure of *TiNb600* and *Ti600* - as observed by AFM (Figure 4) - is suppressed in comparison with samples *TiNb165* and *Ti165*. The surfaces of the HT samples are covered with structure-less patches of TiO_2_ and Nb_2_O_5_. The observed rutile and T-Nb_2_O_5_ phases [4] may increase the biocompatibility of the HT samples relative to the sample in the LT series. Rutile films deposited on PEEK enhanced the adhesion and growth of osteoblasts more than anatase films [29]. It has been suggested that this is induced by an increase in the material surface wettability, and particularly by the presence of acid –OH groups on the rutile films, which attracted Ca^2+^ ions, followed by binding PO_4_^3−^ and by the formation of apatite. Enhanced colonization of rutile with osteoblasts was explained in this way [29]. Thermal treatment of Ti (530–1000°C) increased the content of the rutile phase on the material surface, which contained more densely-packed titanium atoms than the anatase phase, and thus it gave a higher possibility for attachment of hydroxyl groups promoting the formation of calcium phosphate [9].

Amorphous Nb_2_O_5_ phase and anatase are present in the samples of the LT series (Figures 3, 4). According to the argumentation above, this may explain the lower biocompatibility of sample *TiNb165* than of *TiNb600*. Nevertheless, this explanation is questionable: the crystalline phases of *TiNb600* and *Ti600* are at least partially covered by an amorphous oxidic phase, according to AFM (Figure 4). The partial presence of an amorphous phase on the surface of sample *TiNb600* observed in this study is in line with an analysis of its Raman spectrum [4].

The value of ***R_a_*** correlates with the chemical composition of the samples of both the LT series and the HT series. The ***R_a_*** values of *Ti165* and *Ti600* are systematically higher than the ***R_a_*** values of the alloy samples (for numerical values, see Table 2). The correlation of this increase with the adhesion and proliferation of cells supports the idea that there is an optimal range of ***R_a_*** values: it should not be too low (as observed for sample *Nb165*, see the discussion above), but it should be lower than for samples *Ti165* and *Ti600* (Figure 1). Nevertheless, a substantial increase in the ***N^rel^(MG-63)*** of sample *TiNb600* relative to the ***N^rel^(MG-63)*** of sample *TiNb165* (i.e. samples with close values of ***R_a_***) is not in line with this explanation. Moreover, the relative number of MG-63 cells on day 3 of the experiment is higher on the surface of sample *Ti600* (1.51±0.14) than on the surface of sample *Ti165* (1.00±0.29) (cf. Table 3). These effects cannot be related to the morphology and the roughness of the samples.

### Role of the surface charge of the material in cell colonization

Positively and negatively charged sites are present in non-equivalent concentrations on the oxidized surfaces of the Ti, Nb and TiNb samples at physiological conditions [4], [6]. Their distribution on the surface creates a net charge, which is measurable as the ζ-potential [30]. The net charge can be tuned by chemical modification of the surface.

Beneficial effects of both a positive net charge and a negative net charge of artificial materials on the colonization of their surfaces by cells have been admitted (for a review, see [26]). For example, positively-charged Ti surfaces supported the adhesion of fibroblasts more than uncharged Ti [31] and also enhanced the formation of apatite, i.e. an important component of the bone matrix [32]. Similarly, in comparison with uncharged surfaces, the negatively charged surfaces increased the attachment of osteoblasts and fibroblasts to hydrogels [33] or the production of glycosaminoglycans in chondrocytes cultured on hydrogels [34].

Colonization of the artificial material surface by cells is mediated by ECM molecules. The effect of the surface charge of the sample on the interaction with ECM molecules is thoroughly discussed in [35]. ECM molecules may contain charged sites under physiological conditions. For example, the Lys-Arg-Ser-Arg (KRSR) sequence in vitronectin and the Arg-Gly-Asp (RGD) sequence in fibronectin contains deprotonated carboxyl groups and protonated amino-groups under physiological conditions, i.e., they are zwitterions. If the protein is in a suitable conformation, these charged sequences could interact with the surface sites of a material with the opposite charge. The role of the surface charge in the interaction with ECM molecules is certainly more complex than in the explanation given here. However, our simple argument shows that both the positively-charged and the negatively-charged surface sites of Ti (TiNb) can in principle support cell colonization.

According to our previous results, the value of the ζ-potential of *TiNb600* is substantially less negative than the ζ-potential of the surface of *Ti600* (Figure 1d), i.e. the alloy sample contains a larger number of positively charged (basic) sites, possibly due to the presence of defective tetrahedrally coordinated Nb^5+^ species acting as positively-charged Lewis centers [4]. The presence of these surface sites in the alloy sample was used to explain the higher number of osteoblast-like MG-63 cells observed on *TiNb600* than on *Ti600*. A similar but substantially less pronounced effect is observed for sample *TiNb165*. The more apparent increase in cell numbers on sample *TiNb600* than on sample *TiNb165* is not accompanied by a change in the value of the ζ-potential. However, this equality does not mean that the concentration of the positively-charged sites is equal on the surfaces of the two alloy samples.

As two distinct Nb_2_O_5_ and TiO_2_ oxide phases can be distinguished on the surface of alloy samples, the distribution of positively and negatively charged sites on their surfaces can be qualitatively discussed using pertinent samples of pure oxides. The value of the ***ζ-***potential of the alloy sample can be expressed as a linear combination of the ***ζ-***potentials of pure TiO_2_ and Nb_2_O_5_ prepared in the same way. As the ***ζ-***potentials of samples *TiNb165* and *TiNb600* are the same within the experimental error (Table 2, Figure 1d) and the concentrations of Nb are close to each other in these samples (cf. values of ***c(Nb)*** in Table 2), sample *TiNb600* should contain a much higher concentration of positively charged sites related with the Nb_2_O_5_ phase. As the roughness of sample *TiNb600* is similar to the roughness of *TiNb165*, higher colonization by MG-63 cells on sample *TiNb600* than on *TiNb165* can be in accordance with our previous results, related dominantly with the higher concentration of positively-charged sites in the surface Nb_2_O_5_ phase of the HT alloy. This effect is evidently due to the different types of Nb-related sites of the Nb_2_O_5_ phase of the alloy samples. In accordance with these findings, some studies have reported that the positive charge of the material surface was more effective in stimulating cell colonization than the negative charge [31], [36]; for a review, see [26].

The higher number of MG-63 osteoblasts on sample *TiNb600* than on sample *TiNb165* may also be related to the more negative net charge of the TiO_2_ phase. Similarly, there may be a more negative charge associated with the TiO_2_ phase on *TiNb600* than on *TiNb165*. Thus, both positively and negatively charged surface sites of the alloy samples are beneficial for cell colonization but the effect of the negatively-charged sites is less pronounced.

Interestingly, the ζ-potentials of samples *TiNb165* and *Nb165* are the same within the experimental error, while ***N^rel^(x)-TiNb165*** is greater than ***N^rel^(x)-Nb165*** (Table 3, Figure 1). This effect can be attributed to the structural effects of *Nb165*, as discussed above.

Summarizing the above discussion, we propose the relative importance of surface morphology, crystal structure, roughness and charge on the adhesion and proliferation of osteoblast cells on oxidized TiNb alloy. The main factor that caused an increase in the biocompatibility of the alloy samples is their surface charge modulated by surface roughness. Lower roughness of the alloy samples is more beneficial for their colonization by cells. The concentration of both basic and acidic sites influences the value of ***N^rel^(x)-Z***. The value of ***N^rel^(MG-63)Nb165*** is dominantly affected by surface structure effects, while the value of ***N^rel^(MG-63)TiNb600*** is dominantly affected by the distribution of the charge on both the surface TiO_2_ and the Nb_2_O_5_ phases. The effects of surface crystal structure and morphology are less important for the HT alloy sample.

### The effect of cell type on cellular response

The differences in cell colonization among the tested samples were more pronounced when osteoblast-like cells of the Saos-2 line were used instead of MG-63 cells. For example, on day 3 after seeding on *TiNb165* surfaces Saos-2 cells increased their number about 2.55 times compared to polystyrene, while in MG-63 cells, the increase was only 1.77 times (Figures 1a, b; Table 3). This can be attributed to a higher sensitivity of Saos-2 cells to the culture conditions. Ti particles added into the cell culture medium acted cytotoxically on Saos-2 cells and primary human osteoblasts, while the viability of MG-63 cells remained unaffected by Ti particles at any tested dose. This cell behavior was explained by a masking effect of the high proliferative rate of MG-63 cells [14]. The Saos-2 cells proliferated more slowly and reached lower population densities than the MG-63 cells (Tables 3, 4 Figure 1). Similar results were obtained in a study by Saldana *et al.* [14], where the proliferation activity of Saos-2 cells was lower and was closer to primary or low-passage human osteoblasts than the activity in MG-63 cells. In addition, the Saos-2 cells had a better-developed actin cytoskeleton (Figure 5), which was reported to be associated with a less proliferative and more differentiated osteoblast phenotype [37].

MG-63 cells are considered as a good model for studies focused on the initial attachment of cells to various materials, because they express a similar integrin subunit profile to human osteoblasts [15]; for a review, see [16]. In our present study, MG-63 cells also appeared as suitable for studying the formation of focal adhesion plaques in cells on various materials. Vinculin-containing focal adhesion plaques were slightly more developed on Ti and Nb than on TiNb and glass samples (Figure 5). A similar pattern was also observed in mouse osteoblast MC3T3-E1 cells, where distinct vinculin-containing focal adhesion plaques were more apparent on Ti than on TiNb [38].

### Osteogenic cell differentiation on tested samples

Saos-2 cells are generally considered a better model than MG-63 cells for osteogenic cell differentiation, because they display similar activity of alkaline phosphatase (ALP) to that of primary osteoblasts. Saos-2 cells also express and produce higher levels of core binding factor alpha1 (Cbfa1), Osterix (SP7), osteocalcin, bone sialoprotein, decorin and procollagen-I, and show a higher level of mineralization than MG-63 cells [14], [16]. Similar results were also obtained in our study, and were most apparent in the case of ALP mRNA, which was relatively well-expressed in Saos-2 cells, but not in MG-63 cells (Table S1). In addition, the differences in osteogenic cell differentiation among the tested materials were more apparent in Saos-2 cells than in MG-63 cells (Figures 2, 6). In Saos-2 cells, the osteogenic cell differentiation, measured by the expression of osteocalcin and collagen I, showed a trend opposite to the trend for cell proliferation. The mRNA levels for these molecules were higher in cells on *Ti165* and *Nb165* than on the *TiNb165* samples (Figure 2). The levels of ALP mRNA were similar in cells on all three samples, the average value being highest on the *Nb165* samples, i.e. samples with relatively low cell proliferation activity. The ALP mRNA was also higher in cells on samples treated at 165°C than at 600°C; i.e., it was higher on samples with lower cell proliferation activity. This opposite trend of cell proliferation and cell differentiation revealed in our study supports the idea that the restriction of cell proliferation, e.g. by specific physicochemical properties and morphology of the material surface, is a prerequisite for entering the differentiation program [39]; for a review, see [26]. In other words, when the cells invest all their resources and energy in proliferation, they cannot differentiate. In addition, a higher level of osteogenic cell differentiation is associated with better developed cell-matrix contact. Cultivation of human bone marrow cells in an osteogenic cell medium (i.e. supplemented with ascorbic acid, 1,25-dihydroxyvitamin D_3_, 3-glycerol phosphate and dexamethasone) resulted not only in higher expression and synthesis of collagen I, ALP and osteocalcin, but also in the formation of larger and more numerous vinculin-containing focal adhesion plaques [40]. In accordance with this, these plaques were slightly better apparent in cells grown on Ti and Nb than on TiNb, i.e. on samples with higher osteogenic differentiation and lower proliferation activity of the cells (Figure 5).

Not only cell-matrix contacts, but also more intensive cell-cell contacts can help to stop the proliferation of cells and to start the differentiation program. More intensive intercellular contacts are achieved at higher cell densities. Unfortunately, no comparison was made of the cell population densities in the Saos-2 cells on samples treated at 165°C and at 600°C.

Nevertheless, the number of MG-63 cells on the TiNb samples was markedly higher on samples treated at 600°C than at 165°C (Figure 6), and a similar trend can also be assumed for Saos-2 cells. In accordance with this, the expression of osteocalcin and collagen I in Saos-2 cells on samples treated at 600°C was higher than on samples treated at 165°C. In the MG-63 cells, these differences were less pronounced, because the cells are of immature phenotype, are less capable of osteogenic cell differentiation, and they also lack the contact inhibition of growth [16].

It should be pointed out that in comparison with the cells on polystyrene dishes, the expression of osteogenic markers in both cell types on the tested materials increased only slightly, or even decreased. This may be due to the fact that on day 7 the cells still remained in the proliferative phase and were cultured in media without factors promoting osteogenic cell differentiation, such as ascorbic acid, 1,25-dihydroxyvitamin D_3_, 3-glycerol phosphate and dexamethasone [14], [16], [37].

The higher osteogenic cell differentiation on Ti samples could also be supported by the chemical composition of the sample surface, namely the presence of negatively-charged TiO_2_-related sites. The expression of collagen I and osteocalcin was generally higher on Ti than on TiNb, and on samples treated at a higher temperature, where the negative charge is more pronounced. In accordance with this, rat marrow stromal cells cultured on positively charged indium tin oxide showed an increased number of attached cells, but decreased activity of alkaline phosphatase and expression of osteopontin, which were higher on negatively-charged surfaces [41]. Thus, it can be supposed that the adhesion and proliferation of the bone cells in our study is related to the presence of positively-charged (i.e. basic) sites of the alloy Nb_2_O_5_ phase, while osteogenic cell differentiation is related to negatively-charged (acidic) sites of the TiO_2_ phase.

In addition, the higher ***R_a_*** values on the Ti samples (particularly the Ti165 samples) can explain, at least partly, the highest osteogenic differentiation of Saos-2 cells on these samples, manifested by a higher expression of collagen I and osteocalcin. Increased roughness of the material surface has often been reported to promote osteogenic cell differentiation (for a review, see [26]). Increased activity of alkaline phosphatase in mouse osteoblast progenitor MC3T3-E1 cells cultured on Ti treated at 300–750°C was also attributed to the increased surface roughness, together with the increased surface energy and wettability [7].

### Cell immune activation of cells on the tested samples

In our study, we also tested the immune activation of macrophages in contact with the tested materials, because these cells are primary mediators of chronic inflammation and foreign body reaction to the implant [42]. Macrophages are cells of non-specific immune response and help to initiate specific defense mechanisms. This cell type, including macrophage-like cell lines, is a good model for studies on the secretion of pro-inflammatory cytokines, because it is capable of considerably higher secretion of these mediators than osteoblast-like cell lines. In our earlier study, the secretion of TNF-α by MG-63 cells cultured on nanofibrous scaffolds, even after stimulation with bacterial lipopolysaccharide (LPS), was very low, while in murine macrophage-like RAW 264.7 cells, the cytokine secretion was higher by 1 to 2 orders [19]. TNF-α is involved in systemic inflammation and stimulates the acute phase reaction [43]. In this study, we found that hydrothermal treatment at 165°C decreased the immunogenicity of the materials. The secretion of TNF-α by RAW 264.7 cells, grown on the heat-treated materials, into the cell culture medium was significantly lower than the production of TNF-α by these cells on non-treated samples, and was similar to the TNF-α production on standard cell culture polystyrene. In addition, the TNF-α secretion by RAW 264.7 cells on all tested samples was lower by 1 to 2 orders than by these cells after stimulation with bacterial LPS (Table 5). Thus, the cell immune activation on hydrothermally treated Ti, TiNb and Nb can be qualified as very low, and from this point of view, all three materials are suitable for the construction of bone implants. Similar results were obtained on Ti-7Nb-6Al, Ti-13Nb-13Zr and Ti-15Zr-4Nb alloys, treated at 750°C, where the secretion of TNF-alpha by polymorphonuclear leucocytes decreased with increasing temperature. This result was attributed to the enhancement of nanoscale surface roughness by heat treatment, expressed as root mean square roughness (rms). It was equal to 10–20 nm on untreated samples and increased to 100–250 nm on heat-treated samples [44]. The nanostructure of the material surface has also been shown to mitigate the immunogenicity of the material compared to conventional flat materials. For example, the number of adherent macrophages on polyethylene terephthalate coated with ZnO nanorods (rms 31 nm) was reduced in comparison with a substrate coated with flat ZnO films or with microscopic glass coverslips (rms 0.3 nm) [42]. The macrophage movement was restricted on nanostructured titanium (rms 4.76±0.01 nm) compared to flat titanium surfaces (rms 0.37±0.005). Furthermore, macrophages produced lower levels of pro-inflammatory enzyme molecules, cytokines and nitric oxide on the nanostructured titanium [45]. From this point of view, our heat-treated samples also displayed nanoscale surface roughness, having R_a_ from 3.1 to 14.3 nm. On the other hand, microscale surface roughness (R_a_ parameter from 2.59 µm to 4.39 µm) of Ti samples with various surface treatments enhanced the production of TNF-α by RAW 264.7 cells [43].

## Conclusions

We have found that oxidized Ti, TiNb and Nb samples supported the attachment, spreading, proliferation and osteogenic differentiation of human osteoblast-like Saos-2 and MG-63 cells. The proliferation was most pronounced on the TiNb samples, while the Ti samples supported cell differentiation, manifested by a higher expression of collagen I and osteocalcin (the expression of ALP remained similar as on TiNb). On the Nb samples, the cell proliferation and differentiation were comparable to the values on the Ti samples of the LT series. In general, the cell proliferation and differentiation was better on samples treated at 600°C than at 165°C, with the exception of the expression of ALP, which showed an opposite trend. Proliferation of the bone cells seems to be related to the presence of positively-charged (i.e. basic) sites of the alloy Nb_2_O_5_ phase, while cell differentiation was related with negatively-charged (acidic) sites of the alloy TiO_2_ phase. Effects associated with the morphology of the samples appeared less important for their colonization with cells. The differences in cell proliferation and differentiation among the tested materials were more apparent in Saos-2 cells than in MG-63 cells. In addition, the differences in cell proliferation were more apparent on the HT samples, while the differences in osteogenic cell differentiation were more pronounced on the LT samples Heat treatment decreased the cell inflammatory activation on the Ti and TiNb samples, as was revealed by the production of TNF-alpha by macrophage-like RAW 264.7 cells.

## Further Perspectives

Thermal oxidation is a promising surface treatment for Ti and TiNb bone implants. TiNb implants could be used in applications where the stimulation of bone cell growth is needed, e.g. for re-implantation of joint prostheses after their failure, often accompanied by a loss of bone tissue. Ti implants are suitable for application when there is no considerable bone loss, in which case rapid osteogenic cell differentiation is desirable.

Further studies will focus on primary and low passaged osteoblasts, which are a better model of physiological osteoblasts *in situ* than cell lines, and also on long-term studies in order to make a deeper investigation of the aspects of osteogenic cell differentiation.

## Supporting Information

Table S1 Comparison of PCR cycles of cDNA product (ALP) when it reaches the threshold. Seven days of cultivation, MG-63 cells had very low levels of mRNA for ALP, which were at the limit of detection.
(DOC)

Table S2 Levels of mRNA for collagen I and osteocalcin in MG-63 cells (**A**) and for collagen I, osteocalcin and alkaline phosphatase in Saos-2 cells (**B**). Day 7 after seeding on tested materials. Values obtained in cells on metallic samples (Ti, Nb, TiNb) treated at 165°C or 600°C were normalized to the values obtained in cells on polystyrene dishes (PS).
(DOC)

Supporting Information S1 Preparation of the samples.
(DOC)

Supporting Information S2 Quantitative analysis by XPS.
(DOC)

Supporting Information S3 Immunofluorescence staining of vinculin and beta-actin.
(DOC)

Supporting Information S4 Markers of osteogenic cell differentiation.
(DOC)

Supporting Information S5 Measurement of TNF-alpha.
(DOC)
