# Supplementary material for: Interaction of Human Osteoblast-Like Saos-2 and MG-63 Cells with Thermally Oxidized Surfaces of a Titanium-Niobium Alloy
Source: PLoS One. 2014 Jun 30;9(6):e100475. doi: 10.1371/journal.pone.0100475 (PMC4076233; doi:10.1371/journal.pone.0100475)
Supplement: Table S1 — Comparison of PCR cycles of cDNA product (ALP) when it reaches the threshold. Seven days of cultivation, MG-63 cells had very low levels of mRNA for ALP, which were at the limit of detection. (DOC) [file pone.0100475.s001.doc]

**Table S1.** Comparison of PCR cycles of cDNA product (ALP) when it reaches the threshold. Seven days of cultivation, MG-63 cells had very low levels of mRNA for ALP, which were at the limit of detection.

| Sample | ***Ct Saos-2*** | ***Ct MG-63*** |
| --- | --- | --- |
| *Ti165* | 23.62 ± 0.15 | 31.84 ± 0.19 |
| *TiNb165* | 22.39 ± 0.27 | 32.26 ± 0.13 |
| *Nb165* | 23.43 ± 0.19 | 31.86 ± 0.17 |
| *Ti600* | 20.66 ± 0.06 | 29.20 ± 0.86 |
| *TiNb600* | 20.95 ± 0.16 | 30.93 ± 0.25 |

Mean ± S.D. from 2 samples for each experimental group.
