# Supplementary material for: Interaction of Human Osteoblast-Like Saos-2 and MG-63 Cells with Thermally Oxidized Surfaces of a Titanium-Niobium Alloy
Source: PLoS One. 2014 Jun 30;9(6):e100475. doi: 10.1371/journal.pone.0100475 (PMC4076233; doi:10.1371/journal.pone.0100475)
Supplement: Table S2 — Levels of mRNA for collagen I and osteocalcin in MG-63 cells (A) and for collagen I, osteocalcin and alkaline phosphatase in Saos-2 cells (B). Day 7 after seeding on tested materials. Values obtained in cells on metallic samples (Ti, Nb, TiNb) treated at 165°C or 600°C were normalized to the values obtained in cells on polystyrene dishes (PS). (DOC) [file pone.0100475.s002.doc]

**Table S2.** Levels of mRNA for collagen I and osteocalcin in MG-63 cells (**A**) and for collagen I, osteocalcin and alkaline phosphatase in Saos-2 cells (**B**). Day 7 after seeding on tested materials. Values obtained in cells on metallic samples (Ti, Nb, TiNb) treated at 165°C or 600°C were normalized to the values obtained in cells on polystyrene dishes (PS).

A. MG-63 cells

| **mRNA level for:** | **Osteocalcin** | **Collagen I** |
| --- | --- | --- |
| ***Sample*** | **Mean ± SD** | **Mean ± SD** |
| *Ti165* | 0.954 ± 0.0687 | 1.381 ± 0.0332 |
| *Nb165* | 0.941 ± 0.0740 | 1.042 ± 0.140 |
| *TiNb165* | 1.083 ± 0.0811 | 1.144 ± 0.267 |
| *Ti600* | 1.160 ± 0.0827 | 1.484 ± 0.111 |
| *TiNb600* | 1.037 ± 0.516 | 1.355 ± 0.230 |
| *PS* | 1.000 ± 0.118 | 1.000 ± 0.115 |

**B. Saos-2 cells**

| **mRNA level for:** | **Osteocalcin** | **Collagen I** | **Alkaline phosphatase** |
| --- | --- | --- | --- |
| ***Sample*** | **Mean ± SD** | **Mean ± SD** | **Mean ± SD** |
| *Ti165* | 0.923 ± 0.0659 | 1.113 ± 0.0556 | 1.480 ± 0.168 |
| *Nb165* | 0.777 ± 0.0110 | 1.042 ± 0.0209 | 1.786 ± 0.235 |
| *TiNb165* | 0.425 ± 0.0331 ***^PS^*** | 0.667 ± 0.0139 ***^all^*** | 1.591 ± 0.301 |
| *Ti600* | 0.903 ± 0.243 | 1.283 ± 0.130 | 0.935 ± 0.0925 ***^Nb165^*** |
| *TiNb600* | 0.897 ± 0.183 | 1.082 ± 0.221 | 0.939 ± 0.217 ***^Nb165^*** |
| *PS* | 1.000 ± 0.126 | 1.000 ± 0.0802 | 1.000 ± 0.209 ***^Nb165^*** |

Mean ± S.D. from 2 samples for each experimental group. ANOVA, Student–Newman–Keuls Method. Statistical significance: ***^PS, all, Nb165^***: p≤0.05 in comparison with the values obtained in cells on polystyrene dishes, all samples and sample *Nb165*, respectively.
