# Supplementary material for: Interaction of Human Osteoblast-Like Saos-2 and MG-63 Cells with Thermally Oxidized Surfaces of a Titanium-Niobium Alloy
Source: PLoS One. 2014 Jun 30;9(6):e100475. doi: 10.1371/journal.pone.0100475 (PMC4076233; doi:10.1371/journal.pone.0100475)
Supplement: Supporting Information S1 — Preparation of the samples. (DOC) [file pone.0100475.s003.doc]

**Supporting Information S1: Preparation of the samples**

The coupons of β-TiNb alloy were sliced using an SiC cutting wheel from rods prepared by arc-melting 74 at.% Ti (ingot, 99.55%, Frankstahl, Austria) with 26 at.% Nb (ingot, 99.85%, TIC, Brussels, Belgium); diameter of coupons ~10.5 mm, thickness ~1.5 mm. Melting proceeded eight times at 800–1000A/23V with subsequent solution annealing at 850°C for 30 min and quenching in water to achieve the defined homogeneity. The surfaces of the TiNb coupons were polished sequentially with abrasive paper (240, 600, 800, 1000 and 4000 grit) and with a suspension of colloidal SiC (0.05 μm, Colloidal Silicat, Leco) into a mirror-like sheen, using a Buehler machine. The Ti and Nb coupons (9 x 10 mm) cut from foils purchased from Goodfellow Metals, Ltd (Ti 99.6%, thickness 0.7 mm, Nb 99.9%, thickness 1 mm) were polished in the same way.
