# Supplementary material for: Interaction of Human Osteoblast-Like Saos-2 and MG-63 Cells with Thermally Oxidized Surfaces of a Titanium-Niobium Alloy
Source: PLoS One. 2014 Jun 30;9(6):e100475. doi: 10.1371/journal.pone.0100475 (PMC4076233; doi:10.1371/journal.pone.0100475)
Supplement: Supporting Information S2 — Quantitative analysis by XPS. (DOC) [file pone.0100475.s004.doc]

**Supporting Information S2: Quantitative analysis by XPS**

The concentrations *c(x)* of oxygen (***x***=O), titanium (x =Ti), niobium (***x***=Nb) and carbon (***x***=C) of the samples were calculated from the integral intensities *I(x)* of O 1s, Ti 2p, Nb 3d and C 1s photoelectron lines corrected for probability of photoemission [1], transmission function of the analyzer (~E^-1^ [2]) and inelastic mean free path of the photoelectrons (~E^0.5^ [2]). The concentrations were discussed as atomic ratios *a(x)*:

$a(x)=\frac{c(x)}{c(Ti)+c(Nb)}$ (1)

for the alloy samples and

$a(x)=\frac{c(x)}{c(Ti)}$, $a(x)=\frac{c(x)}{c(Nb)}$ (2)

for the Ti and Nb samples). The intensities of the O 1s spectra used for evaluation of *c(O)* were corrected for the presence of oxidized hydrocarbon contamination [3]. The photoelectron line intensities used for evaluating the *a(Nb)* and *a(O)* values of the Nb-containing samples were further corrected for attenuation of the photoelectrons in the layer of hydrocarbon contamination [4]. The estimated *a(O)* values of the samples were expressed relative to the stoichiometric concentration of oxygen (*a(O)****^stechio^)*** as *ΔO*:

*ΔO* = *a****(****O)* – *a(O)****^stechio^*** (3)

The *a(O)****^stechio^*** value of the alloy samples was estimated from *a(Ti)* and *a(Nb)* [3], and nominal values of *a(O)****^stechio^*** were used for the Ti and Nb oxides.

1. Scofield J (1976) Hartree-Slater subshell photoionization cross-sections at 1254 and 1487 eV, J. Electron Spectroscopy Relat Phenom 8: 129-137.
2. Briggs D, Seah MP (1990) Practical surface analysis. Wiley, Chichester.
3. Jirka I, Vandrovcova M, Frank O, Tolde Z, Plsek J, et al. (2013) On the role of Nb-related sites of an oxidized β-TiNb alloy surface in its interaction with osteoblast-like MG-63 cells Materials Sci Eng C 33: 1636-1645.
4. Evans S (1997) Correction for the effects of adventitious carbon overlayers in quantitative XPS analysis. Surf Interface Anal 25: 924-930.
