# Supplementary material for: Interaction of Human Osteoblast-Like Saos-2 and MG-63 Cells with Thermally Oxidized Surfaces of a Titanium-Niobium Alloy
Source: PLoS One. 2014 Jun 30;9(6):e100475. doi: 10.1371/journal.pone.0100475 (PMC4076233; doi:10.1371/journal.pone.0100475)
Supplement: Supporting Information S3 — Immunofluorescence staining of vinculin and beta-actin. (DOC) [file pone.0100475.s005.doc]

**Supporting Information S3: Immunofluorescence staining of vinculin and beta-actin**

The cells were rinsed twice in PBS and fixed with frozen 70% ethanol (room temperature, 20 minutes), pre-treated with 1% bovine serum albumin in PBS containing 0.05% Triton X-100 (Sigma, Cat. No. T6878) for 20 min at room temperature, then treated with 1% Tween (Sigma, Cat. No. P1379) applied for 20 min. The last step was to incubate the cells with primary antibody: mouse monoclonal anti-human vinculin (Sigma, Cat. No. V9131). The antibody was diluted in PBS to a concentration of 1:200 and applied overnight at 4°C. After rinsing with PBS, the secondary antibody, goat anti-mouse F(ab′)2 fragment of IgG (dilution 1:400), was added for 1 h at room temperature. The secondary antibody was conjugated with Alexa Fluor 488 and was purchased from Invitrogen, Molecular Probes (Cat. No. A11017). After incubation with secondary antibody, the cells were rinsed in PBS and incubated with phalloidin (Sigma, Cat. No. P2141) diluted in PBS to a concentration of 1:1000, applied for 30 minutes. The last step was to rinse the samples in PBS. The number of cells on the surface of the material was evaluated on microphotographs taken under an Olympus IX-50 microscope, equipped with an Olympus DP 70 digital camera, and on an SP2 confocal microscope (Leica, Germany).
