# Supplementary material for: Interaction of Human Osteoblast-Like Saos-2 and MG-63 Cells with Thermally Oxidized Surfaces of a Titanium-Niobium Alloy
Source: PLoS One. 2014 Jun 30;9(6):e100475. doi: 10.1371/journal.pone.0100475 (PMC4076233; doi:10.1371/journal.pone.0100475)
Supplement: Supporting Information S4 — Markers of osteogenic cell differentiation. (DOC) [file pone.0100475.s006.doc]

**Supporting Information S4: Markers of osteogenic cell differentiation**

Collagen I and ALP are usually classified as early markers of osteogenic cell differentiation [1], but some studies consider collagen I as an early marker and ALP as a middle marker [2]. In accordance with this, in primary cultures of fetal rat calvarial osteoblasts, collagen I was expressed during the proliferative phase, i.e. during the first 10-12 days after isolation, while ALP was expressed later, i.e. after the decline in proliferation, i.e. on days 12-18 after isolation [3]. Osteocalcin as a late or terminal marker of osteogenic cell differentiation was not detectable before day 12, and reached considerable levels only after day 20 [3]. However, in osteosarcoma cell lines, such as Saos-2 and MG-63 cells, osteocalcin and also ALP were detectable earlier, even on days 1-7 after seeding on both mRNA and protein level [4].

1. Born A-K, Rottmar M, Lischer S, Pleckova M, Bruinink A, et al. (2009) Correlation cell architecture with osteogenesis: First steps towards live single cell monitoring. Eur Cells Mater 18: 49-62.
2. Gong Z, Wezeman FH (2004) Inhibitory effect of alcohol on osteogenic differentiation in human bone marrow-derived mesenchymal stem cells. Alcohol Clin Exp Res 28: 468-479.
3. Stein GS, Lian JB, Owen TA (1990) Relationship of cell growth to the regulation of tissue-specific gene expression during osteoblast differentiation. The FASEB J 4: 3111-3123.
4. Saldana L, Bensiamar F, Bore A, Vilaboa N (2011) In search of representative models of human bone-forming cells for cytocompatibility studies. Acta Biomater 7: 4210-4221.
