# Supplementary material for: Interaction of Human Osteoblast-Like Saos-2 and MG-63 Cells with Thermally Oxidized Surfaces of a Titanium-Niobium Alloy
Source: PLoS One. 2014 Jun 30;9(6):e100475. doi: 10.1371/journal.pone.0100475 (PMC4076233; doi:10.1371/journal.pone.0100475)
Supplement: Supporting Information S5 — Measurement of TNF-alpha. (DOC) [file pone.0100475.s007.doc]

**Supporting Information S5: Measurement of TNF-alpha**

The measurement was performed using a commercially available Mouse TNF-α ELISA Kit (Thermo Scientific Inc., Rockford, IL, USA, Cat. No. 1347.4 EMTNFA) according to the manufacturer’s protocol. Cells stimulated with lipopolysaccharide (LPS; Sigma, USA, Cat. No. L 2654, concentrations of 1 ng/mL, 10 ng/mL, and 50 ng/mL) were used as a positive control for TNF-α production. Cells growing on PS were exposed to LPS on day 6 and then cultured for an additional 24 hours. As a negative control, we used RPMI-1640 medium supplemented with fetal bovine serum, which was not exposed to cells. Absorbances were measured using an ELISA Versa Max Microplate Reader at 450 and 550 nm. Three independent samples for each experimental group were used and were measured twice. The concentrations of TNF-α were expressed in pg per 1,000,000 cells, as the numbers of cells grown on the samples and on PS differed from each other.
